# Geographic variations in ACGME PGY‐2 PM&R residency program salaries compared to cost of living

**DOI:** 10.1002/pmrj.70154

**Published:** 2026-05-09

**Authors:** Jimmy Wen, Shannon Dwyer, Megan Kou, Apple Zhu, Arsh Alam, Naomi Pai, Jennifer S. Addleman, Joshua Levin

**Affiliations:** ^1^ California Northstate University College of Medicine Elk Grove California USA; ^2^ University of California, Berkeley Berkeley California USA; ^3^ University of California, Davis Davis California USA; ^4^ Department of Orthopaedic Surgery and Neurosurgery Stanford University Stanford California USA; ^5^ Sutter Roseville Roseville California USA

## INTRODUCTION

A resident's lifestyle is shaped by many factors, including income, workload intensity, challenges in maintaining work–life balance, emotional stressors, and continuous educational demands.^1^ Residency salaries are largely determined by institutional graduate medical education policies, which are primarily funded by Medicare, particularly through the Centers for Medicare and Medicaid Services.^2^ A sum payment is provided to hospitals, which is then allocated to residents at the institution's discretion. There are limited data on the methodology used by institutions to determine resident salaries.

According to the American Association of Medical Colleges^3^ 2024 survey of resident stipends, the average postgraduate year‐2 (PGY‐2) resident salary was ∼$68,901, which is similar to the average U.S. salary of $67,920 reported by the U.S. Bureau of Labor Statistics.^4^ Prior studies evaluating internal medicine, diagnostic radiology, and ophthalmology resident salaries compared to regional cost of living (COL) demonstrated wide variability and no correlation.^5^, ^6^, ^7^ This regional disparity is especially important for specialties where training begins at the PGY‐2 level, such as physical medicine and rehabilitation (PM&R), as trainees in advanced programs have to consider additional financial burdens, including moving expenses if the preliminary program location is far from their advanced program.^8^ No study has directly evaluated PGY‐2 salaries across U.S. PM&R residency programs compared to their regional COL.

The objective of this study is to compare PGY‐2 salaries across U.S. PM&R residency programs relative to geographic COL indices. These data may be used to provide applicants with more information to make informed choices for their careers, which may influence quality of life, stress, and overall training satisfaction. Although program location is often the most important factor when applying,^7^, ^9^ salary has historically been ranked lower in priority.^9^ Nevertheless, the financial considerations of salary relative to COL are an important consideration during training.

## METHODS

### Study design

This study was a cross‐sectional analysis of PM&R PGY‐2 salary and COL for all Accreditation Council for Graduate Medical Education (ACGME)‐accredited PM&R residency programs. Program salary data for the 2024–2025 academic year were obtained from publicly available information on 107 of the 113 individual program websites. For the six programs where the 2024–2025 salaries were not available, the most recent adjacent years were calculated (three from 2023 to 2024 and three from 2025 to 2026). Only the base PGY‐2 salary was obtained as supplemental compensation from bonuses, housing stipends, and other aspects of a resident compensation package were excluded for consistency and comparability across programs.

### Data collection

The annual COL was calculated using the Massachusetts Institute of Technology (MIT) Living Wage Calculator's hourly wage (for one adult, no children) and 2080 hours per year, reflecting the standardized full‐time work schedule used by the U.S. government.^10^ The Living Wage Calculator estimates the annual income required to live in a given county. This COL is based on food, housing, transportation, income taxes, and other civic expenses within that given county, which is viewed as the minimum wage for meeting the basic needs for one adult. Although residents typically work longer hours, the 40‐hour work week was used to estimate baseline living costs, assuming full‐time employment. Notably, for the two Puerto Rico programs, the MIT Living Wage Calculator does not provide estimates, and the COL was estimated using the Numbeo data instead. The monthly cost of living for one adult was extracted and multiplied by 12 to obtain the annual value. To maintain consistency with the MIT Living Wage Calculator, the annual cost was divided by 2080 hours to convert into an hourly equivalent.

The surplus income was determined as the PGY‐2 salary minus the annual COL. Surplus percentage was determined by dividing the surplus income by the PGY‐2 salary. The geographical region of the programs (West, Midwest, Northeast, Southeast, Southwest) was categorized by the U.S. Census Bureau classification system.^11^ The two Puerto Rico programs were excluded from the regional comparison analysis as they do not fall within the U.S. Census geographic regions.

### Statistical analysis

Descriptive statistics were performed for PGY‐2 salary, COL, and surplus income. One‐way analysis of variance (ANOVA) was computed to assess differences in COL and surplus income percentage across regions, with an effect size (*η* ^2^) calculated to assess the variation attributable to regional differences.

A multiple linear regression was used to assess the relationship between PGY‐2 salary, COL, and surplus income, with geographic region as a categorical variable using dummy coding, where the West was used as the reference group. This allowed for adjusting the COL to assess salary differences across regions. Correlation analysis was conducted to assess the relationship between PGY‐2 salary and COL, surplus percentage and COL, surplus percentage and PGY‐2 salary. Adjusted *R* ^2^, F‐statistic, and *p* values were reported. All statistical analyses and figure/table creation were performed with Microsoft Excel (Microsoft Office Version 16.80, 2023).

## RESULTS

### Geographic overview

PGY‐2 salaries ranged from $49,000 to $96,241, COL ranged from $13,499 to $68,328, and surplus income percentage ranged from −5% to 46%. The average PGY‐2 salary, COL, surplus income, and surplus income percentage, as well as the maximum and minimum surplus income, are presented in Table 1. The full details for the programs are found in Table S1.

**TABLE 1:** Average PGY‐2 salary, cost of living, surplus income, and percentage.

Average PGY‐2 salary ($)	70,766
Average cost of living ($)	50,112
Average surplus income ($)	20,653
Average surplus (%)	29.2
Max surplus income ($)	35,759
Min surplus income ($)	−2522

PGY‐2, postgraduate year‐2.

Regional analysis revealed considerable variation in both income and expenses, which influenced surplus income. The average salary across the regions ranged from $64,769 (Southeast) to $78,306 (West), COL from $45,316 (Midwest) to $57,521 (West), and surplus income from $15,456 (Southeast) to $24,750 (Midwest). These comparisons are detailed in Table 2 and illustrated in Figure 1.

**TABLE 2:** Average PGY‐2 salary, cost of living, surplus income, and percentage by region.

Region	Avg PGY‐2 salary ($)	Avg cost of living ($)	Avg surplus income ($)	Avg surplus (%)
Northeast	75,818.06	54,919.73	20,898.33	0.2757
Midwest	70,066.03	45,316	24,750.03	0.3523
Southeast	64,768.77	49,312.34	15,456.43	0.2315
Southwest	68,120.13	46,436	21,684.13	0.3179
West	78,306.07	57,520.67	20,785.41	0.2603

PGY‐2, postgraduate year‐2.

**FIGURE 1:**
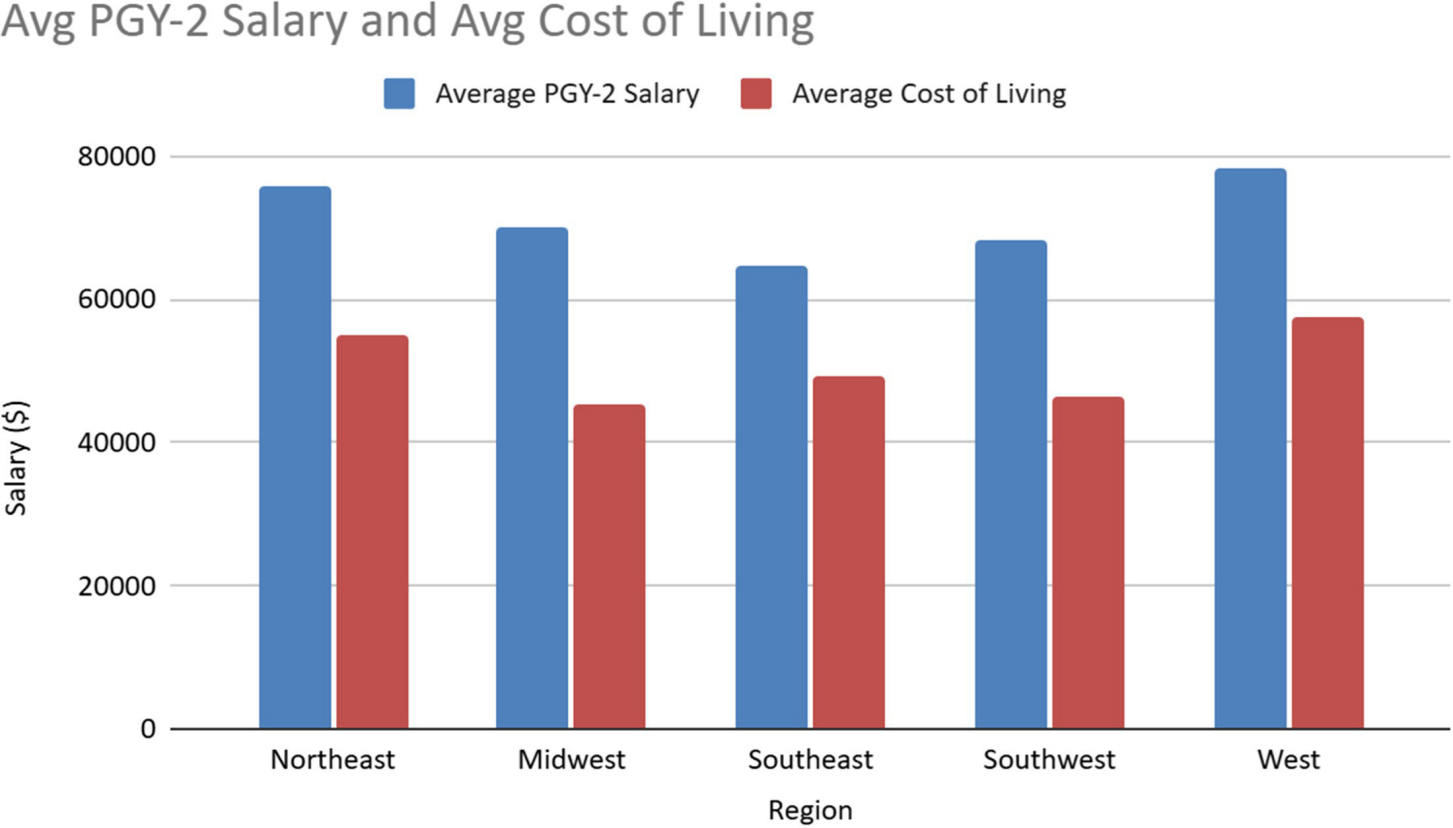
Average PGY‐2 salary and average cost of living. PGY‐2, postgraduate year‐2.

### Surplus income by region

Surplus income as a percentage of salary also varied by region, as shown in Figure 2.

**FIGURE 2:**
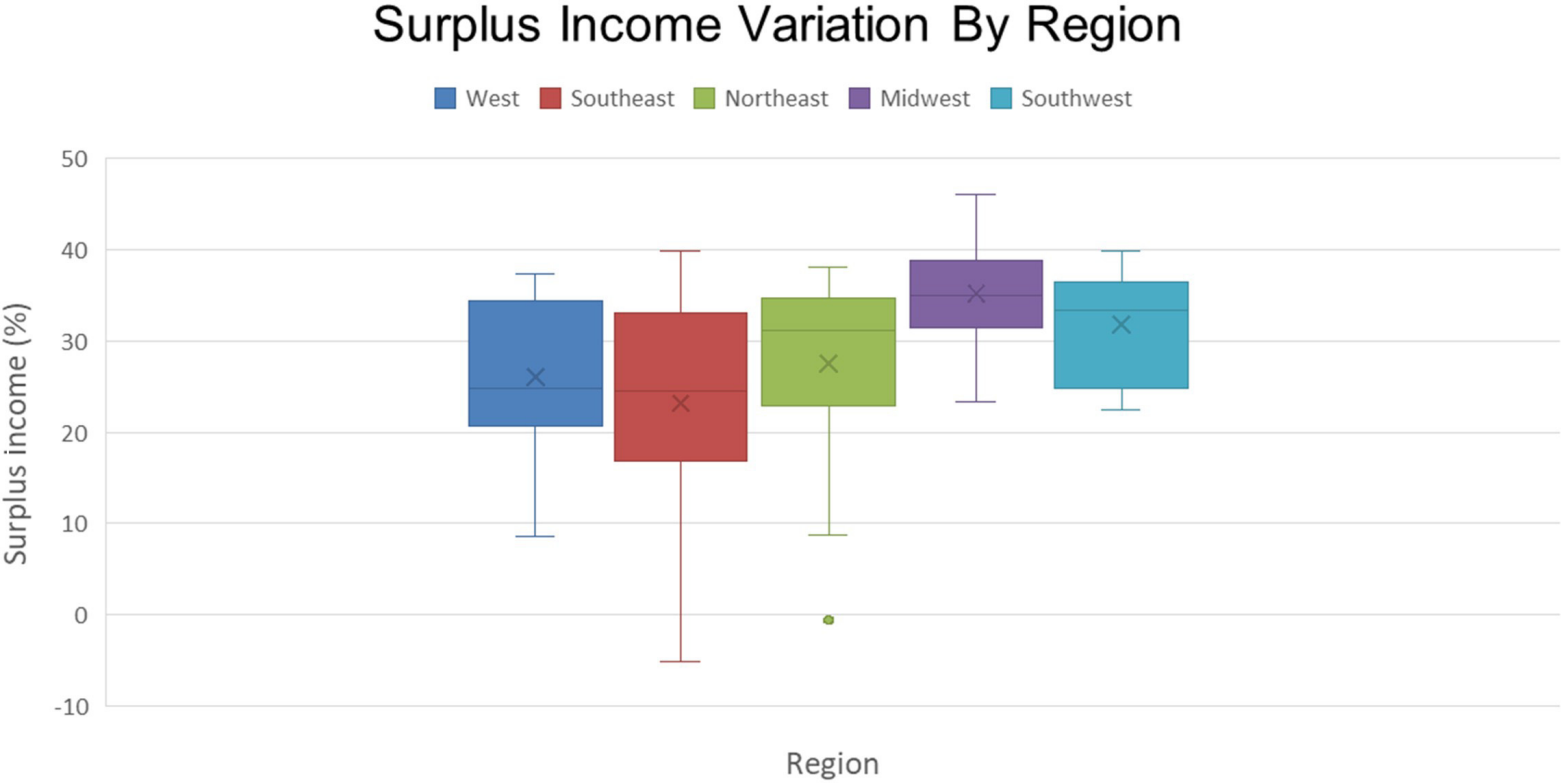
Regional variation in PGY‐2 surplus income. × inside the box: Mean surplus income, Central line inside the box: median surplus income, Box boundaries: (IQR)—25th and 75th percentiles, Whiskers (brackets): minimum and maximum values within 1.5 × IQR quartiles, Point beyond the whiskers: outliers. IQR, interquartile range; PGY‐2, postgraduate year‐2.

The Midwest had the highest average surplus percentage at 35.2%, and the Southeast had the lowest at 23.1%. A wide range of surplus percentages was observed, ranging from deficits of −5% to surpluses of 46%. The full distribution is shown in Figure 3.

**FIGURE 3:**
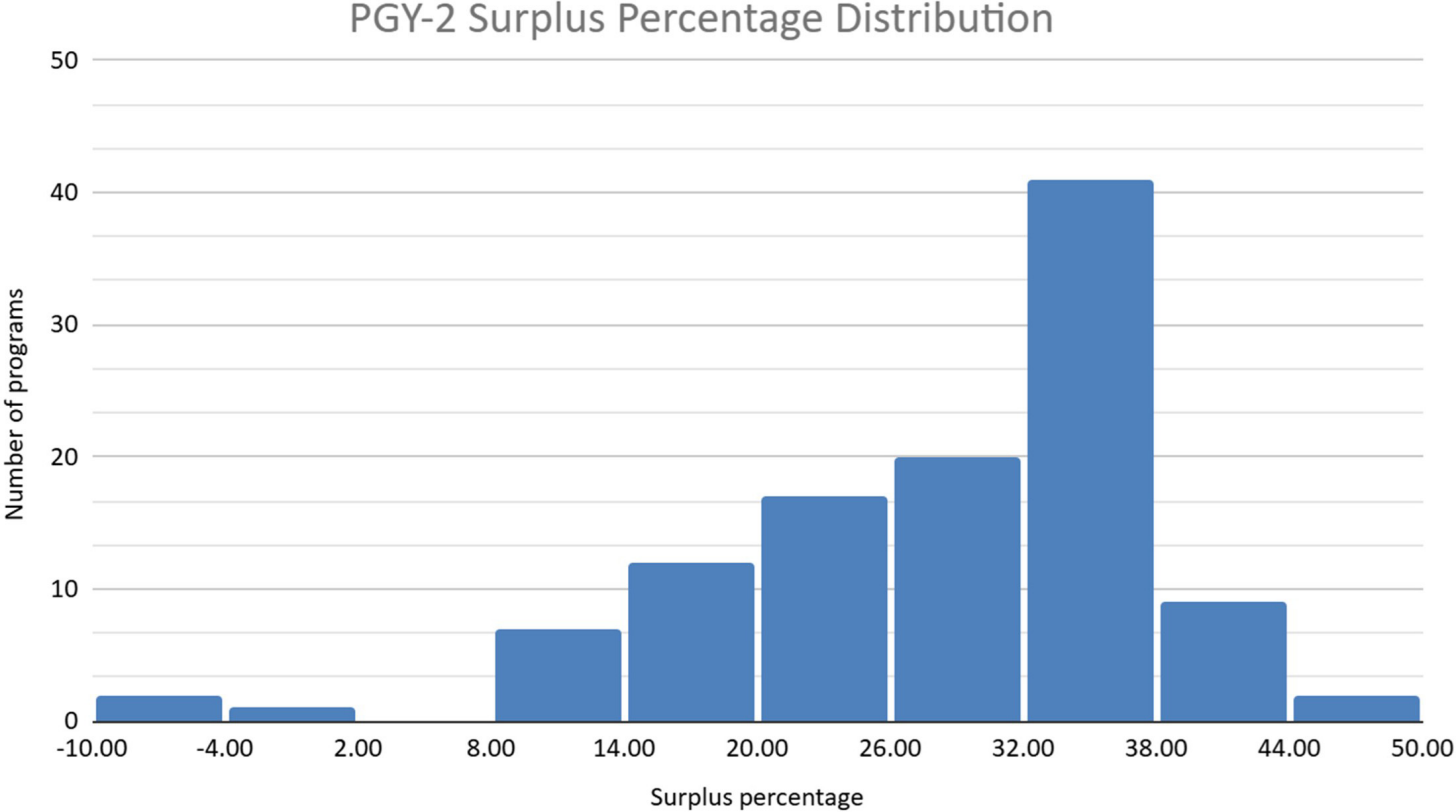
Distribution of PGY‐2 surplus percentages.PGY‐2, postgraduate year‐2.

### Statistical analysis

Surplus income percentage varied significantly across U.S. regions, as demonstrated by a one‐way ANOVA (*F*(4,108) = 6.65, *p* < .001). There was also a large effect size (*n* ^2^ = 0.201), with ∼20% of the variance in surplus income percentage being attributable to regional differences (Table S2).

Correlation analysis demonstrated a moderate positive relationship between PGY‐2 salary and COL (*r* = 0.58). A moderate negative correlation was observed between surplus income percentage and COL (*r* = −0.64). Additionally, a weak positive correlation was found between surplus percentage and PGY‐2 salary (*r* = 0.25).

To further explore these relationships, a multiple linear regression was performed to assess the effects of annual COL and geographic region on surplus income percentage across 111 PM&R programs (excluding the two Puerto Rico programs). The model was statistically significant (adjusted *R* ^2^ = 0.539, *p* < .001), with COL emerging as a strong negative predictor of surplus percentage. Among regional comparisons, whereas programs in the West demonstrated a significantly higher COL compared to those in the Southeast (*p* < .001), no other statistically significant differences were found in regional comparisons. Full regression results are presented in Table S3.

## DISCUSSION

Comparing salary to COL within each geographic region rather than analyzing salary alone allows for the measurement of purchasing power in each region and may reveal disparities between residents in high and low COL areas. These regional disparities may carry implications for residents' financial stress, quality of life, and program geographic recruitment patterns. PGY‐2 salary had a moderate positive correlation with COL (*r* = 0.58), suggesting that programs located in higher COL areas may tend to offer higher salaries. However, surplus percentage had a more moderately negative correlation with COL (*r* = −0.64), indicating that residents in higher‐cost areas likely retain a smaller portion of their income after expenses. There was also a weak positive correlation for surplus percentage and PGY‐2 salary (*r* = 0.25), implying that higher salaries do not consistently result in proportionally higher financial surplus. The surplus income varied significantly across the five U.S. regions (one‐way ANOVA, *p* < .001) with a large effect size (*n* ^2^ = 0.2), indicating that geographic region had a substantial impact on surplus income for residents. These findings underscore the financial disparities faced by residents based on geographic location, suggesting that residents in the Southeast may experience the greatest economic burden, and those in the Midwest may retain the largest portion of their income after living expenses.

Although PGY‐2 salaries appeared higher in regions with increased COL, they did not equate to increased resident purchasing power. Notably, programs in the Southeast had the lowest average surplus percentage, and the Midwest had the highest. The West and Northeast regions had the highest average PGY‐2 salary and COL. However, our findings also support the notion that applicants should not rule out a location based solely on high COL. For example, several West and Northeast programs in counties with some of the highest COL had equivalent or higher surplus income.

This research supports other studies identifying disparities between COL and resident salaries. Over the past 2 decades, resident salaries have not kept up with the rising COL, with inflation‐adjusted PGY‐1 salaries decreasing by 0.23% while rental prices increased by 17.8%.^12^ This disparity was similarly seen in residency salary trends from 2014–2015 to 2021–2022, which found that salary/COL did not significantly change over this study period.^1^ This directly affects resident family planning, as demonstrated by a 2025 cross‐sectional analysis of PGY‐1 to PGY‐7 resident salaries for 2022–2023 that found that single‐parent families, two‐adult (one child, single‐income), two‐adult (one child, dual‐income), and families with more than one child earned a living wage at PGY‐7, PGY‐5, PGY‐3, and never, respectively.^13^ Interestingly, unionization was not associated with increased affordability for residents.^13^ Our study supports these findings of a consistent mismatch between resident salaries and COL. Altogether, this suggests that salary structures have not correlated with COL, highlighting the need for future evaluation of compensation strategies.

Another important factor for residents is housing affordability. Brewster et al.'s study evaluated housing affordability for the 2022–2023 year (855 programs) and found that residents at 511 (59.8%) programs were rent‐burdened (local Zillow Observed Rent Index >30% of gross monthly income), 246 programs (28.8%) offered moving allowances, and 117 (13.7%) provided housing stipends.^12^ The majority of residents who were rent burdened attended programs that were the least likely to offer housing benefits. Similar to other studies, housing unaffordability varied significantly with geographic region and urbanicity.^12^ These findings reflect the real‐world financial challenges that residents face and align with our MIT Living Wage‐based analyses, which account for housing costs.

### Limitations

These findings must be considered in the context of their limitations. First, the MIT Living Wage Calculator estimates COL using standardized models and may not reflect COL at the individual level. Intraregional and interregional nuances and urban/suburban/rural cost of living differences may not be fully reflected. For example, academic programs and associated hospitals are typically located in urban areas, and residents are typically expected to live within 30 minutes of the hospital if they are on call. Thus, although the surrounding county may contain suburban or rural areas with lower COL, residents may be expected to live in high COL areas within a given county due to their resident responsibilities. Future studies can use zip‐code adjustments in their analyses or perform spatial modeling to provide a more precise estimate of COL. Additionally, institutional differences, such as the presence of unions, hospital size, and other institutional factors, can also be explored.

Second, the PGY‐2 salary was obtained through publicly available information and assumed to represent base salary only. Notably, a small number of programs (*n* = 6) had salary data from 2023–2024 or 2025–2026 academic years, which introduces slight temporal variations in salary that can affect comparative analyses. Additionally, although residents are generally ineligible for bonuses, many residents may receive supplemental income through moonlighting, housing stipends, meal allowance, relocation reimbursement, educational funds (study material/travel/conferences, research), or personal financial endeavors. Some of these funds are subject to income tax, whereas others are reimbursed expenses directly from the funding institution. Thus, program‐specific stipends and benefits were not incorporated into the analysis due to inconsistent reporting, precluding their inclusion in a standardized and consistent comparison across PM&R programs. This may have led to an underestimation of the total financial package available to PM&R residents. Future studies can contact programs for information on stipends to compare this adjusted income to COL.

Third, this analysis was based on the COL of one adult without children, which does not represent residents who are in single‐income households with dependents or dual‐income households with or without dependents.

Fourth, this analysis did not account for variations in federal, state, or local income taxes across locations. State‐level income tax policies vary substantially: some states (eg, Nevada, Texas, and Florida) do not impose state income taxes, whereas others (eg, California, New York) have higher state income tax rates. Therefore, the usable disposable income may differ from the surplus incomes calculated in this study.

Fifth, this study did not stratify based on program affiliation, such as university based, community based with university affiliation, and community based. This may influence salary decisions, funding sources, and reflect differing institutional structures.

## CONCLUSION

PGY‐2 salaries, COL, and surplus income between 2024 and 2025 (with a small proportion from 2023 to 2024 and 2025 to 2026) across the 113 included ACGME PM&R resident programs varied significantly across geographical regions. Programs in the Midwest provided the highest average surplus income, whereas those in the Southeast had the lowest. These findings highlight disparities in resident salary compared to COL and can provide valuable information to aid applicants' decision‐making process when choosing a residency program.

## FUNDING INFORMATION

All authors declare no funding was received.

## DISCLOSURE

Dr Levin reports consulting fees from Limber Technologies, Scilex Pharmaceuticals, State Farm Insurance Company, Allstate Insurance Company, Liberty Mutual Insurance Company, Geico Insurance Company, Hanover Insurance Company. No other authors had disclosures.

## Supporting information


**Table S1.** Postgraduate year‐2 (PGY‐2) salary, cost of living (COL), and surplus income broken down by program.

**Table S2.** Analysis of variance of postgraduate year‐2 (PGY‐2) surplus income.

**Table S3.** Regression statistics.

## Data Availability

The datasets used and/or analyzed in the current study are available upon reasonable request. Please contact J.W. to request data from the study.
