# Supplementary material for: Geographic variations in ACGME PGY‐2 PM&R residency program salaries compared to cost of living
Source: PM R. 2026 May 9;18(11):1352–7. doi: 10.1002/pmrj.70154 (PMC13768730; doi:10.1002/pmrj.70154)
Supplement: Supplementary file 1 [file PMRJ-18-1352-s001.docx]

| **Program Name** | **County** | **State** | **Region** | **PGY-2 Salary (USD)** | **Living Wage (Hourly, 1 Adult No Children)** | **Annual Cost of Living (Hourly x 2080)** | **Surplus Income (Salary - COL)** | **Surplus %** |
| --- | --- | --- | --- | --- | --- | --- | --- | --- |
| **Program 1** | **Jefferson** | **Alabama** | **Southeast** | **65,469** | **21.43** | **44574.4** | **20,895** | **0.319152576** |
| **Program 2** | **Maricopa** | **Arizona** | **Southwest** | **69,000** | **25.73** | **53518.4** | **15,482** | **0.2243710145** |
| **Program 3** | **Pulaski** | **Arkansas** | **Southeast** | **63,640** | **20.28** | **42182.4** | **21,458** | **0.3371715902** |
| **Program 4** | **San Bernardino** | **California** | **West** | **76,481.60** | **27.69** | **57595.2** | **18,886.40** | **0.2469404406** |
| **Program 5** | **Los Angeles** | **California** | **West** | **74,209.26** | **27.81** | **57844.8** | **16,364.46** | **0.2205177629** |
| **Program 6** | **Los Angeles** | **California** | **West** | **91,754** | **27.81** | **57844.8** | **33,909** | **0.3695664494** |
| **Program 7** | **Orange** | **California** | **West** | **88,227.23** | **32.2** | **66976** | **21,251.23** | **0.2408692872** |
| **Program 8** | **Los Angeles** | **California** | **West** | **66,560** | **27.81** | **57844.8** | **8,715** | **0.1309375** |
| **Program 9** | **San Mateo** | **California** | **West** | **96,241.60** | **34.76** | **72300.8** | **23,940.80** | **0.2487572941** |
| **Program 10** | **Sacramento** | **California** | **West** | **85,319.70** | **26.76** | **55660.8** | **29,658.90** | **0.3476207722** |
| **Program 11** | **Arapahoe** | **Colorado** | **West** | **82,169.48** | **26.71** | **55556.8** | **26,612.68** | **0.3238754827** |
| **Program 12** | **Hartford** | **Connecticut** | **Northeast** | **75,006** | **23.55** | **48984** | **26,022** | **0.3469322454** |
| **Program 13** | **New Haven** | **Connecticut** | **Northeast** | **79,602** | **24.97** | **51937.6** | **27,664** | **0.3475339816** |
| **Program 14** | **N/A** | **District of Columbia** | **Southeast** | **73,500** | **29.25** | **60840** | **12,660** | **0.172244898** |
| **Program 15** | **Manatee** | **Florida** | **Southeast** | **69,343** | **21.74** | **45219.2** | **24,124** | **0.3478909191** |
| **Program 16** | **Broward** | **Florida** | **Southeast** | **58,516** | **25.31** | **52644.8** | **5,871** | **0.1003349511** |
| **Program 17** | **Alachua** | **Florida** | **Southeast** | **69,444** | **21.61** | **44948.8** | **24,495** | **0.3527331375** |
| **Program 18** | **Miami-Dade** | **Florida** | **Southeast** | **49,000** | **24.77** | **51521.6** | **-2,522** | **-0.05146122449** |
| **Program 19** | **Broward** | **Florida** | **Southeast** | **64,688** | **25.31** | **52644.8** | **12,043** | **0.1861736334** |
| **Program 20** | **Duval** | **Florida** | **Southeast** | **75,093** | **22.49** | **46779.2** | **28,314** | **0.3770497916** |
| **Program 21** | **Miami-Dade** | **Florida** | **Southeast** | **64,151.14** | **24.77** | **51521.6** | **12,629.54** | **0.1968716378** |
| **Program 22** | **Pensacola** | **Florida** | **Southeast** | **64,140** | **22.24** | **46259.2** | **17,881** | **0.2787776738** |
| **Program 23** | **Miami-Dade** | **Florida** | **Southeast** | **49,000** | **24.77** | **51521.6** | **-2,522** | **-0.05146122449** |
| **Program 24** | **Hillsborough** | **Florida** | **Southeast** | **69,343** | **24.27** | **50481.6** | **18,861** | **0.2720014998** |
| **Program 25** | **Fulton** | **Georgia** | **Southeast** | **65,716** | **26.5** | **55120** | **10,596** | **0.161239272** |
| **Program 26** | **Cook** | **Illinois** | **Midwest** | **76,902** | **24.42** | **50793.6** | **26,108** | **0.3395022236** |
| **Program 27** | **Cook** | **Illinois** | **Midwest** | **72,738** | **24.42** | **50793.6** | **21,944** | **0.3016910006** |
| **Program 28** | **Cook** | **Illinois** | **Midwest** | **71,134.68** | **24.42** | **50793.6** | **20,341.08** | **0.2859516624** |
| **Program 29** | **DuPage** | **Illinois** | **Midwest** | **76,902** | **27.66** | **57532.8** | **19,369** | **0.251868612** |
| **Program 30** | **Allen** | **Indiana** | **Midwest** | **67,168** | **21.23** | **44158.4** | **23,010** | **0.3425678895** |
| **Program 31** | **Marion** | **Indiana** | **Midwest** | **68,700** | **20.48** | **42598.4** | **26,102** | **0.3799359534** |
| **Program 32** | **Wyandotte** | **Kansas** | **Midwest** | **67,608** | **21.43** | **44574.4** | **23,034** | **0.3406934091** |
| **Program 33** | **Fayette** | **Kentucky** | **Southeast** | **63,059** | **19.68** | **40934.4** | **22,125** | **0.350855548** |
| **Program 34** | **Jefferson** | **Kentucky** | **Southeast** | **64,179.69** | **21.55** | **44824** | **19,355.69** | **0.3015859067** |
| **Program 35** | **Orleans Parish** | **Louisiana** | **Southeast** | **56,653** | **20.78** | **43222.4** | **13,430** | **0.2370641274** |
| **Program 36** | **Baltimore City** | **Maryland** | **Northeast** | **70,322** | **21.15** | **43992** | **26,330** | **0.3744205227** |
| **Program 37** | **Baltimore City** | **Maryland** | **Northeast** | **66,083** | **21.15** | **43992** | **22,091** | **0.334291724** |
| **Program 38** | **Suffolk** | **Massachusetts** | **Northeast** | **81,635** | **32.11** | **66788.8** | **14,846** | **0.1818607215** |
| **Program 39** | **Suffolk** | **Massachusetts** | **Northeast** | **86,667** | **32.11** | **66788.8** | **19,878** | **0.229362964** |
| **Program 40** | **Washtenaw** | **Michigan** | **Midwest** | **77,609** | **23.82** | **49545.6** | **28,063** | **0.3615998145** |
| **Program 41** | **Wayne** | **Michigan** | **Midwest** | **61,989** | **20.39** | **42411.2** | **19,578** | **0.3158270016** |
| **Program 42** | **Kent** | **Michigan** | **Midwest** | **62,439** | **23.01** | **47860.8** | **14,578** | **0.2334790756** |
| **Program 43** | **Ingham** | **Michigan** | **Midwest** | **77,609** | **20.12** | **41849.6** | **35,759** | **0.4607635712** |
| **Program 44** | **Oakland** | **Michigan** | **Midwest** | **67,117** | **22.25** | **46280** | **20,837** | **0.3104578572** |
| **Program 45** | **Wayne** | **Michigan** | **Midwest** | **67,177** | **20.39** | **42411.2** | **24,766** | **0.3686648704** |
| **Program 46** | **Hennepin** | **Minnesota** | **Midwest** | **68,901** | **22.98** | **47798.4** | **21,103** | **0.3062742195** |
| **Program 47** | **Olmsted** | **Minnesota** | **Midwest** | **75,093** | **22.06** | **45884.8** | **29,208** | **0.3889603558** |
| **Program 48** | **Boone** | **Missouri** | **Midwest** | **68,169** | **19.3** | **40144** | **28,025** | **0.4111106221** |
| **Program 49** | **St. Louis** | **Missouri** | **Midwest** | **72,940** | **19.61** | **40788.8** | **32,151** | **0.4407896902** |
| **Program 50** | **Douglas** | **Nebraska** | **Midwest** | **67,504** | **21.92** | **45593.6** | **21,910** | **0.3245792842** |
| **Program 51** | **Clark** | **Nevada** | **West** | **62,858** | **24.1** | **50128** | **12,730** | **0.2025199656** |
| **Program 52** | **Middlesex** | **New Jersey** | **Northeast** | **74,866.21** | **26.03** | **54142.4** | **20,723.81** | **0.276811261** |
| **Program 53** | **Gloucester** | **New Jersey** | **Northeast** | **66673.53** | **26.2** | **54496** | **12177.53** | **0.1826441468** |
| **Program 54** | **Essex** | **New Jersey** | **Northeast** | **76,045** | **24.94** | **51875.2** | **24,170** | **0.3178354921** |
| **Program 55** | **Bernalillo** | **New Mexico** | **Southwest** | **67,676** | **20.82** | **43305.6** | **24,370** | **0.3601040251** |
| **Program 56** | **Albany** | **New York** | **Northeast** | **73,652** | **23** | **47840** | **25,812** | **0.3504589149** |
| **Program 57** | **Bronx** | **New York** | **Northeast** | **76,930** | **25.48** | **52998.4** | **23,932** | **0.3110828025** |
| **Program 58** | **Kings** | **New York** | **Northeast** | **87,014.74** | **29.25** | **60840** | **26,174.74** | **0.300808116** |
| **Program 59** | **Kings** | **New York** | **Northeast** | **79,386** | **29.25** | **60840** | **18,546** | **0.2336180183** |
| **Program 60** | **Nassau** | **New York** | **Northeast** | **61,000** | **29.5** | **61360** | **-360** | **-0.005901639344** |
| **Program 61** | **Nassau** | **New York** | **Northeast** | **83,600** | **29.5** | **61360** | **22,240** | **0.2660287081** |
| **Program 62** | **New York** | **New York** | **Northeast** | **90,011** | **32.85** | **68328** | **21,683** | **0.2408927798** |
| **Program 63** | **New York** | **New York** | **Northeast** | **77,000** | **32.85** | **68328** | **8,672** | **0.1126233766** |
| **Program 64** | **New York** | **New York** | **Northeast** | **81,769** | **32.85** | **68328** | **13,441** | **0.1643776981** |
| **Program 65** | **New York** | **New York** | **Northeast** | **98,700** | **32.85** | **68328** | **30,372** | **0.3077203647** |
| **Program 66** | **Suffolk** | **New York** | **Northeast** | **81,881** | **30.36** | **63148.8** | **18,732** | **0.2287734639** |
| **Program 67** | **Monroe** | **New York** | **Northeast** | **76,000** | **22.82** | **47465.6** | **28,534** | **0.3754526316** |
| **Program 68** | **Monroe** | **New York** | **Northeast** | **73,424** | **22.82** | **47465.6** | **25,958** | **0.3535410765** |
| **Program 69** | **Nassau** | **New York** | **Northeast** | **79,500** | **29.5** | **61360** | **18,140** | **0.2281761006** |
| **Program 70** | **Onondaga** | **New York** | **Northeast** | **73,509** | **22.59** | **46987.2** | **26,522** | **0.3607966371** |
| **Program 71** | **Westchester** | **New York** | **Northeast** | **74,283** | **32.60** | **67808** | **6,475** | **0.08716664647** |
| **Program 72** | **Westchester** | **New York** | **Northeast** | **74,450** | **32.60** | **67808** | **6,642** | **0.08921423774** |
| **Program 73** | **Orange** | **North Carolina** | **Southeast** | **66,936** | **25.95** | **53976** | **12,960** | **0.1936177842** |
| **Program 74** | **Mecklenburg** | **North Carolina** | **Southeast** | **68,224** | **26.59** | **55307.2** | **12,917** | **0.1893292683** |
| **Program 75** | **Pitt** | **North Carolina** | **Southeast** | **68,491** | **19.81** | **41204.8** | **27,286** | **0.3983910295** |
| **Program 76** | **Geauga** | **Ohio** | **Midwest** | **70,444** | **22** | **45760** | **24,684** | **0.3504059963** |
| **Program 77** | **Hamilton** | **Ohio** | **Midwest** | **70,387** | **19.81** | **41204.8** | **29,182** | **0.4145964454** |
| **Program 78** | **Cuyahoga** | **Ohio** | **Midwest** | **67,564** | **20.33** | **42286.4** | **25,278** | **0.374128234** |
| **Program 79** | **Cuyahoga** | **Ohio** | **Midwest** | **69,117** | **20.33** | **42286.4** | **26,831** | **0.3881910384** |
| **Program 80** | **Franklin** | **Ohio** | **Midwest** | **68,439** | **22.24** | **46259.2** | **22,180** | **0.3240812987** |
| **Program 81** | **Lucas** | **Ohio** | **Midwest** | **63,258** | **19.28** | **40102.4** | **23,156** | **0.3660501439** |
| **Program 82** | **Columbia** | **Pennsylvania** | **Northeast** | **68,307** | **20.34** | **42307.2** | **26,000** | **0.3806315605** |
| **Program 83** | **Dauphin** | **Pennsylvania** | **Northeast** | **69,371** | **22.22** | **46217.6** | **23,153** | **0.3337619466** |
| **Program 84** | **Philadelphia** | **Pennsylvania** | **Northeast** | **71,238** | **23.26** | **48380.8** | **22,857** | **0.3208568461** |
| **Program 85** | **Philadelphia** | **Pennsylvania** | **Northeast** | **74,799** | **23.26** | **48380.8** | **26,418** | **0.3531892138** |
| **Program 86** | **Philadelphia** | **Pennsylvania** | **Northeast** | **73,008** | **23.26** | **48380.8** | **24,627** | **0.3373219373** |
| **Program 87** | **Philadelphia** | **Pennsylvania** | **Northeast** | **76,953.44** | **23.26** | **48380.8** | **28,572.64** | **0.3712977613** |
| **Program 88** | **Allegheny** | **Pennsylvania** | **Northeast** | **71,307** | **22.76** | **47340.8** | **23,966** | **0.3360988402** |
| **Program 89** | **Lackawanna** | **Pennsylvania** | **Northeast** | **60,250** | **20.74** | **43139.2** | **17,111** | **0.2839966805** |
| **Program 90** | **Berks** | **Pennsylvania** | **Northeast** | **69,389.15** | **22.15** | **46072** | **23,317.15** | **0.3360345241** |
| **Program 91** | **San Juan Municipality** | **Puerto Rico** | **Other** | **43,412** | **6.49** | **13499.2** | **29,913** | **0.6890445038** |
| **Program 92** | **San Juan Municipality** | **Puerto Rico** | **Other** | **43,412** | **6.49** | **13499.2** | **29,913** | **0.6890445038** |
| **Program 93** | **Charleston** | **South Carolina** | **Southeast** | **63,101** | **26.04** | **54163.2** | **8,938** | **0.1416427632** |
| **Program 94** | **Charleston** | **South Carolina** | **Southeast** | **62,858** | **26.04** | **54163.2** | **8,695** | **0.1383244774** |
| **Program 95** | **Davidson** | **Tennessee** | **Southeast** | **63,461** | **25.41** | **52852.8** | **10,608** | **0.1671609335** |
| **Program 96** | **Davidson** | **Tennessee** | **Southeast** | **74,986** | **25.41** | **52852.8** | **22,133** | **0.2951644307** |
| **Program 97** | **Travis** | **Texas** | **Southwest** | **67,628** | **24.42** | **50793.6** | **16,834** | **0.2489264802** |
| **Program 98** | **Dallas** | **Texas** | **Southwest** | **68,045** | **23.06** | **47964.8** | **20,080** | **0.2951017709** |
| **Program 99** | **Dallas** | **Texas** | **Southwest** | **69,431** | **23.06** | **47964.8** | **21,466** | **0.3091731359** |
| **Program 100** | **Tarrant** | **Texas** | **Southwest** | **64,000** | **23.18** | **48214.4** | **15,786** | **0.24665** |
| **Program 101** | **Harris** | **Texas** | **Southwest** | **70,086** | **21.65** | **45032** | **25,054** | **0.357475102** |
| **Program 102** | **Harris** | **Texas** | **Southwest** | **70,085** | **21.65** | **45032** | **25,053** | **0.3574688679** |
| **Program 103** | **Lubbock** | **Texas** | **Southwest** | **65,165** | **18.84** | **39187.2** | **25,978** | **0.3986465127** |
| **Program 104** | **Bexar** | **Texas** | **Southwest** | **70,085** | **20.84** | **43347.2** | **26,738** | **0.381505315** |
| **Program 105** | **Salt Lake** | **Utah** | **West** | **75,529** | **24.27** | **50481.6** | **25,047** | **0.3316262628** |
| **Program 106** | **Albemarle** | **Virginia** | **Southeast** | **66,379** | **25.77** | **53601.6** | **12,777** | **0.1924916013** |
| **Program 107** | **Norfolk** | **Virginia** | **Southeast** | **65,774** | **22.43** | **46654.4** | **19,120** | **0.2906862894** |
| **Program 108** | **Richmond City** | **Virginia** | **Southeast** | **65,341** | **23.45** | **48776** | **16,565** | **0.253516169** |
| **Program 109** | **King** | **Washington** | **West** | **70,160** | **30.82** | **64105.6** | **6,054** | **0.08629418472** |
| **Program 110** | **Spokane** | **Washington** | **West** | **70,163** | **21.11** | **43908.8** | **26,254** | **0.3741886749** |
| **Program 111** | **Monongalia** | **West Virginia** | **Southeast** | **63,040** | **20.17** | **41953.6** | **21,086** | **0.3344923858** |
| **Program 112** | **Dane** | **Wisconsin** | **Midwest** | **71,758** | **22.46** | **46716.8** | **25,041** | **0.3489673625** |
| **Program 113** | **Milwaukee** | **Wisconsin** | **Midwest** | **73,050** | **20.09** | **41787.2** | **31,263** | **0.4279644079** |

**Table S1: PGY-2 Salary, COL, and surplus income broken down by program**
