# Supplementary material for: Geographic variations in ACGME PGY‐2 PM&R residency program salaries compared to cost of living
Source: PM R. 2026 May 9;18(11):1352–7. doi: 10.1002/pmrj.70154 (PMC13768730; doi:10.1002/pmrj.70154)
Supplement: Supplementary file 2 [file PMRJ-18-1352-s002.docx]

| Multiple R | 0.7486 |
| --- | --- |
| R Square | 0.5604 |
| Adjusted R Square | 0.5395 |
| Standard Error | 0.0676 |
| Observations | 111 |

**Table S3: Regression Statistics**
