# Supplementary material for: Geographic variations in ACGME PGY‐2 PM&R residency program salaries compared to cost of living
Source: PM R. 2026 May 9;18(11):1352–7. doi: 10.1002/pmrj.70154 (PMC13768730; doi:10.1002/pmrj.70154)
Supplement: Supplementary file 3 [file PMRJ-18-1352-s003.docx]

| Sources of Variation | SS | df | MS | F | p-value |
| --- | --- | --- | --- | --- | --- |
| Between Groups | 0.219 | 4 | 0.547 | 6.649 | <0.0001 |
| Within Groups | 0.872 | 106 | 0.008 | - | - |
| Total | 1.091 | 110 | - | - | - |

**Table S2: ANOVA of PGY-2 Surplus Income**

**SS: sum of squares, df: degrees of freedom, MS: mean square (SS/df), F: test statistic**
